# New Appliances and Things Medical

**Published:** 1895-04-20

**Authors:** 


### NEW APPLIANCES AND THINGS MEDICAL.

### CALVERT'S CARBOLISED TOW, TEN PER CENT.

A good many years back we noticed this tow very favourably, and our extended experience of it enables us to repeat that as a dressing for foul wounds there is nothing better. It is soft, elastic, comfortable to the patient, and the percentage of carbolic acid in it provides a real disinfectant and deodorant for the discharge. The elasticity seems to exert in some cases a most beneficial effect on indolent foul ulcers, causing them to take on a more rapid healing than when the same immediate dressing was used, but with a plain bandage next to it and no tow between.

